# Safety of exercise training in multiple sclerosis: a protocol for an updated systematic review and meta-analysis

**DOI:** 10.1186/s13643-021-01751-0

**Published:** 2021-07-20

**Authors:** Y. C. Learmonth, L. A. Pilutti, M. P. Herring, R. W. Motl, B. Chan, A. P. Metse

**Affiliations:** 1 Discipline of Exercise Science, Murdoch University, Murdoch, WA Australia; 2 Perron Institute for Neurological and Translational Science, Perth, WA Australia; 3 Centre for Molecular Medicine and Innovative Therapeutics, Health Futures Institute, Murdoch University, Murdoch, WA Australia; 4 Centre for Healthy Ageing, Health Futures Institute, Murdoch University, Murdoch, WA Australia; 5 Interdisciplinary School of Health Sciences, Brain and Mind Research Institute, University of Ottawa, Ottawa, Canada; 6 Department of Physical Education and Sport Sciences, University of Limerick, Limerick, Ireland; 7 Physical Activity for Health Research Cluster, Health Research Institute, University of Limerick, Limerick, Ireland; 8 Department of Physical Therapy, School of Health Professions, University of Alabama at Birmingham, Birmingham, AL USA; 9 University Library, Murdoch University, Murdoch, WA Australia; 10 Discipline of Libraries, Archives, Records & Information Science, School of Media, Creative Arts & Social Inquiry, Faculty of Humanities, Curtin University, Perth, WA Australia; 11 Discipline of Psychology, School of Health and Behavioural Sciences, University of the Sunshine Coast, Sippy Downs, QLD Australia; 12 School of Psychology, Faculty of Science, University of Newcastle, Callaghan, NSW Australia

**Keywords:** Multiple sclerosis, Exercise-training, Relapse, Adverse event, Serious adverse event

## Abstract

**Background:**

There has been an exponential growth in the number of clinical research studies regarding exercise training in multiple sclerosis, and literature reviews and meta-analyses have documented the many benefits of exercise training. This research further requires careful review for documenting the safety of exercise training in multiple sclerosis, as clarity on safety represents a major hurdle in the clinical prescription of exercise behaviour.

**Objectives:**

To enhance understanding of the feasibility of exercise in multiple sclerosis, we (1) provide a protocol of a systematic review and meta-analysis that summarises rates and risks of clinical relapse, adverse events (i.e., an unfavourable outcome that occurs during the intervention delivery time period), and serious adverse events (i.e., an untoward occurrence that results in death or is life threatening, requires hospitalisation, or results in disability during the intervention delivery time period), as well as retention, adherence, and compliance, from randomised controlled trials of exercise training in persons with multiple sclerosis; and (2) identify moderators of relapse, adverse events, and serious adverse event rates.

**Methods:**

Eight field-relevant databases will be searched electronically. Studies that involve a randomised controlled trial of exercise training (with non-exercise, non-pharmacological, comparator), report on safety outcomes, and include adults with multiple sclerosis will be included. Rates and relative risks of the three primary outcomes (relapse, adverse event, and serious adverse event) will be calculated and reported each with standard error and 95% confidence interval. Random-effects meta-analysis will estimate mean population relative risk for outcomes. Potential sources of variability, including participant characteristics, features of the exercise stimulus, and comparison condition, will be examined with random-effects meta-regression with maximum likelihood estimation.

**Discussion:**

The results from this systematic review and meta-analysis will inform and guide healthcare practitioners, researchers, and policymakers on the safety of exercise training in persons with multiple sclerosis. Where possible, we will identify the impact of exercise type, exercise delivery style, participant disability level, and the prescription of exercise guidelines, on the safety of exercise training. The result will identify critical information on the safety of exercise in persons with multiple sclerosis, while also identifying gaps in research and setting priorities for future enquiries.

**Systematic review registration:**

PROSPERO 2020 CRD42020190544

**Supplementary Information:**

The online version contains supplementary material available at 10.1186/s13643-021-01751-0.

## Background

Multiple sclerosis (MS) is a chronic, immune-mediated disease of the central nervous system (CNS) with no known cure [1]. There has been an estimated 10% increase in the global prevalence of MS in the last two decades [2], with estimates indicating MS now affects over 2.8 million people globally [3], with one million cases in the USA alone [4]. MS is the most common non-traumatic disabling disease among young adults and is more common in females [5]. The underlying cause of the disease is unknown, and the disease follows a relapsing-remitting clinical trajectory in the majority of cases. MS commonly results in cumulative and heterogeneous physical and cognitive disability, as well as debilitating symptoms such as fatigue, depression, and pain [6]. The health burden is high such that the 2016 Global Burden of Disease study reported that over 1.1 million disability-adjusted life-years were attributable to MS [2].

The burden of MS has both personal and societal impacts, and this extends into participation in optimal health behaviours, such as exercise participation. Upwards of 80% of persons with MS do not engage in sufficient amounts of exercise necessary for health-related benefits [7]. Exercise training represents the salient approach to symptom management, and safe approaches to increase and maintain exercise practices should be promoted among persons with MS throughout the disease trajectory [8, 9].

Optimal health and wellness are of high priority for people with MS [10, 11], and persons with MS can achieve optimal health through positive health behaviours, particularly participation in exercise. Exercise is commonly included in rehabilitation programmes addressing the functional impairments associated with MS [12]; exercise is widely considered to be an essential strategy in the clinical management of MS [13]. Over three decades of scientific enquiry have established the many health benefits of exercise training in persons with MS [14]. Systematic reviews and meta-analysis have concluded that exercise training can improve physical fitness and walking mobility [15, 16], balance [17], cognition [18], fatigue [19], depressive symptoms [20–22], and quality of life [19]. There is evidence indicating that exercise has a positive effect on the hippocampus [23, 24], sleep quality [25, 26], and cardiovascular and metabolic comorbidity [27, 28]. The volume and quality of research output on exercise in MS populations has increased substantially in recent years [9].

There further is a shift in the clinical landscape surrounding exercise prescription in MS. This is based on the first clinical exercise guidelines for persons with mild-to-moderate MS that were published in 2013 [19, 29], and have now been updated and expanded [30]. Importantly, these guidelines have been endorsed by international MS societies [8, 31], yet we still know very little about the safety profile of exercise among persons with MS. This is particularly important, as healthcare providers have seemingly been reluctant in prescribing exercise training for clinical management of MS [32], with evidence indicating that this may, in part, be because there is limited comprehensive understanding of the risk profile of this behaviour [13]. The recent surge in exercise training studies in MS [9] further underscores the need to identify the safety profile on this salient intervention in MS.

Relapse risk, adverse events (AE), and serious adverse events (SAE) [33, 34] indicate the construct of safety in MS clinical study, and we describe these terms in Table 1. To date, only three reviews have considered the safety of exercise training for MS [35–37]. The most comprehensive included 26 RCTs published up until November 2013 [35]. That study reported that people with MS receiving a form of exercise intervention had lower rates of relapse equating to approximately 27% *lower* relapse rate for exercise training versus non-exercise control conditions. Notably, the rate of other AE across studies was higher in the exercise as represented by a 67% higher risk of AE from exercise. In terms of AE, 22 of the 26 RCTs recorded dropout rate, and this is important as another indicator of intervention feasibility; the mean dropout rate across studies was 15.2% for control group participants and 15.5% for exercise training participants.

**Table 1:** Items for extraction and definition of terms used in this systematic review

	Extractions format and definitions
**Study details**	
Author	First author surname
Year	Year published
Title	Title of publication
Country	Corresponding author affiliation
Funding source	From text
Conflict of interest declared	Reported as yes, no, other
Clinical trial registry ID	From text
**Methods**	
Study design	RCT, cluster RCT, other
Study aims	From text
Years of study recruitment	Years
Retention strategy employed	Described where relevant as remumeration or other method
**Participants**	
Age	Mean (standard deviation), median (range)
Gender	Male, female, other/unspecified
Race/ethnicity	Described where possible from text
Socioeconomic status	Employment status, income, level of education, or similar
MS type	Type of MS of participants; relapse remitting, primary progressive, secondary progressive, progressive relapsing
Years diagnosed with MS	Mean (standard deviation), median (range)
Disability level*	Described where possible using Expanded Disability Status Scale or Patient Determined Disease Steps. Means (std) or median (range) will be reported. Other descriptors of disability will be reported. Described where possible as mild, moderate, or severe disability. Mild disability is usually categorised as EDSS <4.5 or PDDS 0–3, moderate disability is usually categorised as EDSS 4.5–6 or PDDS 4–5, and severe disability is usually categorised as EDSS 6.5–9.5 or PDDS 7–8.
Inclusion/exclusion criteria	From text
**Intervention**	
Frequency	Sessions/Wk
Intensity (aerobic exercise)	Described where relevant as light, moderate, vigorous Light intensity exercise is usually between 9 and 11 on the Borg 6 to 20 RPE scale, or 1–2 on the Borg 1–10 RPE scale. Alternatively, light intensity exercise is 30–39% VO_2_R or HRR [60]. Vigorous intensity exercise is usually between 14 and 17 on Borg’s 6–20 RPE scale. Alternatively, vigorous intensity exercise is 60–89% VO_2_R or HRR [60]
Type*	Described where possible as aerobic, resistance, flexibility, balance, neuromotor, combined, aerobic interval training, or other Aerobic exercise training is a type of exercise in which the body’s large muscles move rhythmically for sustained periods [61]. Minimal guidelines for aerobic exercise are two 30-min sessions per week [19, 30]. Resistance exercise training refers to activities where muscles work or hold against an applied force or weight to improve muscular fitness; traditional resistance training incorporates progressions and rest intervals [62, 63]. Minimal guidelines for resistance exercise are two sessions per week comprising 5–10 exercises [19, 30]. Flexibility exercise training considers activities that are designed to preserve or extend range of motion [61]. Balance training refers to activities designed to increase lower body strength and reduce the likelihood of falls [61]. Neuromotor or multicomponent exercise training combines different motor skills (e.g., balance, coordination, gait, agility, and proprioceptive training) [62, 64]; this is not combined exercise training. Combined exercise is a combination of different exercise types within an intervention (e.g., aerobic exercise and resistance exercise). Aerobic interval involves varying the exercise intensity at fixed time interval during a single exercise session [60].
Session time	Session min/d
Exercise prescription	Described where possible as modality of exercise, equipment, sets and repetition, and rest periods. Detail of progression through programme will be identified.
Meeting minimum guidelines dose*	Identified from the frequency of aerobic exercise (2/wk) and resistance exercise (2/wk) sessions, the intensity and time of aerobic exercise (moderate intensity, 30 min [19, 29]), and the intensity of resistance exercise (one to four sets of 10 to 15 repetitions at 10–15 repetitions maximum [19, 29])
Programme duration	Number of weeks
Facilitator qualifications and training	Described where possible according to clinical qualification and/or studying qualification
Mode of delivery*	Described where possible as supervised, independent, or remotely supervised Supervised programmes are in person and supervised by a researcher trained in exercise rehabilitation, an allied healthcare professional, or students trained in exercise rehabilitation on allied healthcare. We will extract data on the setting where the exercise training is supervised. Independent programmes are completed in the participant’s community or home, and a researcher or health professional does not supervise the intervention in real-time. Information may be provided via mail or asynchronously via telehealth. Participants may provide feedback on intervention adherence to the researchers/health professionals. Synchronous communication is limited between the researcher/health professional team and the participant. Remotely supervised programmes are completed in the participant’s community or home; asynchronous telecommunication to provide supervision, programming, or intended advice is an important study construct. We will extract data on the setting where the exercise training is supervised.
Description of comparator	Control condition will be categorised. We will extract data on the instruction provided to control participants, example categories include “usual activity”, “usual activity + social programme”, “education”
**Primary outcomes of interest**	Only events occurring during the intervention period will be considered
Relapse	Relapse is an acute onset of new or worsening neurological symptoms, lasting over 24 h [65] Will be reported on using terms “relapse” or a combination of words pertaining to “increase symptoms”, “symptom exacerbation”. From text, distinction of increased symptoms indicating a relapse will be determined from the text.
Adverse event* (Adverse effects)	An adverse event is an unfavourable outcome that occurs during or after the intervention [33]; we consider AE to have a causal relationship, or not, to the intervention. We will focus on events that occur within the intervention delivery time-period (e.g., the weeks the intervention is delivered). Will be reported on terms “adverse event”, “adverse effect”, or “injury”, “illness”, “*falls*”*,* “*joint pain*”*,* “*upper respiratory tract infection*”*,* “*sprains*”*,* “*strains*”*,* “*muscle pain*”*,* “*symptom exacerbation*” Described where possible as musculoskeletal, respiratory illness, fall, cardiovascular, other From text, distinction between adverse event and adverse effect will be determined from text. We will identify the presence of causal language for example “engagement in intervention led to…” or “event was unrelated to participation in the intervention” to assist in our identification of adverse effects.
Serious adverse event* (Serious adverse effects)	A SAE is an untoward occurrence that results in death or is life-threatening, requires hospital admission, or results in significant or permanent disability that occurs during or after the intervention [34]; we consider SAEs with a causal relationship, or not, to the intervention. We will focus on events that occur within the intervention delivery time-period (e.g., the weeks the intervention is delivered). Will be reported on terms “serious adverse event”, “heart attack”, “myocardial infarction”, “stroke”, “pulmonary embolism”, “fracture”, and “dislocation” to assist in our identification of adverse effects. Described where possible as musculoskeletal, respiratory illness, fall, cardiovascular From text, distinction between serious adverse event and serious adverse effect will be determined from text. We will identify the presence of causal language for example “engagement in intervention led to…”
Retention rates	Retention is the completion of outcome measurements following the intervention. Will be reported on number completed first post-intervention follow-up data collection/number recruited
Intervention adherence rate	Adherence is the extent to which the participant follows the intervention corresponding with the agreed recommendations of the study [66]; we consider adherence as attendance to exercise sessions. Will be reported on number of attended exercise sessions for the intervention. From text: terms of attendance to exercise sessions for the intervention, e.g., “attendance”, “journal”, “diary”, aspects reported will include “Frequency”, “intensity”, “modality”, “duration”
Intervention compliance rate	Compliance is the extent to which the participant exercise behaviour matches the agreed recommendations of the study [66], we consider compliance as the completion of the prescribed exercise programme. Will be reported on compliance and completion of the prescribed programme. From text: terms of completion of the exercise prescription, e.g., “completed”, “dose”, “sets”, “repetitions”, “prescription”
**Risk of bias**	
*PEDro* [49]	
Inclusion criteria and source	Not scored—extracted as above
Random allocation	Yes/no
Concealed allocation	Yes/no
Baseline comparability	Yes/no
Subject blinding	Yes/no
Therapist blinding	Yes/no
Assessor blinding	Yes/no
Completeness of follow-up	Yes/no
Intention to treat analysis	Yes/no
Between group statistical comparisons	Yes/no
Point measures and variability	Yes/no

Note: *VO*_*2*_*R* VO_2_ reserve, *HRR* heart rate reserve, *EDSS* Expanded Disability Status Scale, *PDDS* patient-determined disease steps

*Considered for subgroup analyses

Another review included safety outcomes as a sub-analysis during a review of the efficacy of physical interventions in MS [36]. That systematic review included three additional studies to the focal review in 2014, and authors provided a narrative commentary of AE, relapse, and dropout rate. One more recent systematic review focused on high-intensity aerobic interval training in MS, and inclusive of studies up to September 2017 noted the reporting of no AE in six of the seven included studies [37]. However, the authors did not provide an analytical review of safety constructs. One other systematic review and meta-analysis of AE reported AE and SAE across 16 different health conditions, with MS investigated as part of the *Neurological* subgroup [38]. The authors established that in neurological conditions, there is a decreased risk of non-serious AE when participating in exercise training interventions. Our review in 2014 identified a need for more transparent recording and reporting of safety outcomes in MS exercise training studies [39], and we highlighted a lack of transparent reporting of exercise training protocols and variability in exercise prescriptions. Our contemporaries recently noted that reporting of training protocols is improving [36]. However, those researchers did not take analytical approaches to compare safety between and across exercise interventions. There has been no clear distinction between SAE and non-serious AE. Such distinctions are of high importance as we judge the severity of any event and focus on the likely attribution exercise training may have on relapse, SAE, or AE in MS. A review of safety in randomised controlled exercise training studies in MS will allow fair comparison, and our planned review will focus on relapse, SAE, and AE as primary outcomes.

Today, alongside the increasing number of clinical exercise trials in MS [9], there has been a move towards exercise prescription at vigorous intensities, through the medium of interval training [37] (defined in Table 1), as an example. There is an urgent need to build on what is already known about the safety of exercise in MS, particularly to closely focus on the contemporary research published after that important review. To date, the focus on intervention characteristics, such as exercise type, delivery style (e.g., supervised, independent, or remotely supervised), participant disability level (e.g., mild, moderate, or severe, as determined via, e.g., the Expanded Disability Status Scale [1]), or the prescription of exercise consistent with minimal exercise guidelines for persons with MS [19, 29], have not been considered in terms of safety. Establishing the safety profile of exercise interventions at this level will offer greater clarity in intervention delivery and assist with providing data on the feasibility of the exercise guidelines.

The comprehensive review of the safety of exercise training must further include information on retention rates, alongside adherence to attending the intervention and compliance with the prescribed exercise of the intervention. Recently, one study has taken a meta-analytical approach to identify adherence and study completion rates in 41 MS exercise training studies. That study identified a pooled estimate of adherence at 0.73 (CI 0.68–0.78) when including participants who did not complete the study [40]. The authors indicated that further work is warranted to identify the influencing factors on retention and adherence; our review will provide essential data on the relationship between outcomes of safety and retention, adherence, and compliance to exercise interventions, and therefore, address the request of these authors.

The proposed systematic review builds on our previous review considering publications from 2013 onward to (a) quantify the relative risk of relapses, AE, SAE, and dropout (i.e., retention rate) for exercise conditions compared to non-exercise comparison conditions; and (b) provide a proxy measure of participation with exercise training among persons with MS by considering intervention retention, adherence, and compliance. Secondary aims will include exploration of potential sources of variability in overall relative risk, including variables of theoretical, practical, and/or prior empirical relation to exercise interventions, such as (c) exercise types, (d) exercise delivery styles, and (e) disability levels (defined in Table 1 and based on established disability cut-points [41]), and (f) prescription of exercise training consistent with the minimal exercise guidelines for persons with mild to moderate MS [19, 29], and we will examine year of publication as a variable in the overall effect. We will further identify the reporting of rates of relapse, rates of adverse effects (refer to Table 1, these are AE directly reported as attributable to the intervention), serious adverse effects (refer to Table 1, these are AE directly reported as attributable to the intervention), retention, adherence to attending the exercise training, and compliance with the prescribed exercise training in randomised controlled trials.

## Methods

The protocol will adhere to the Preferred Reporting Items for Systematic review and Meta-Analysis Protocol) statement [42]. An original review by the research team [35] was a basis for the current review. The protocol for the review is registered with the International Prospective Register of Systematic Reviews (PROSPERO 2020 CRD42020190544). As detailed in the authors’ previously published systematic reviews [43–45], standard methods will be used for data extraction, data synthesis and meta-analysis, and meta-regression analysis. In addition, the Participants, Interventions, Comparisons, and Outcomes (PICO) strategy was adapted to suit the scope of the review and used to guide the search strategy [46]. In brief, and described in detail in the search strategy and Table 1, the terms were persons with *Multiple Sclerosis* (Participants) and *Exercise training* (Intervention). The comparison is non-exercise and non-pharmacological comparator, and relapse, AE, and SAE are the main outcomes.

### Eligibility criteria

The inclusion criteria are as follows: a randomised controlled trial (RCT) design with reporting of safety outcomes overall and per condition during the study; adults (aged 18 years or above) with multiple sclerosis; an intervention type which meets the definition of exercise training [47]. All non-active/non-exercise control conditions will be considered, for example, “usual activity, non active education programme”. Further, data derived from articles included in our original review [35] will be included.

The following exclusion criteria will be applied: studies not written in English, non-human participants, physical interventions which are primarily sedentary (e.g., manual therapy, breathing exercises, pelvic floor exercises), control interventions which are pharmacology based.

### Search strategy and information sources

A university librarian will assist the research team in the development of the search strategy, and this librarian will undertake the database searches. The following databases will be searched for reports of trials of exercise training in persons with MS: Ovid MEDLINE All, Ovid Embase, ProQuest PsycINFO, Cochrane Central Register of Controlled Trials (CENTRAL), Ebsco CINAHL, Scopus, Web of Science Core Collection (CC), and PEDro–Physiotherapy Evidence. All databases are recommended as core database for our field on inquiry [48].

Search terms will include free text keywords and relevant subject headings, and there will be no language restrictions in the automated search (we will remove non-English language articles during article screening). All databases will be searched from 2013 to present, following completion of the search in November 2013 for our original review. As the relevant search engines do not allow a search of exact months, we will hand search any relevant publications from 2013 to ascertain if published in November/December 2013. Supplementary material 1 comprises the proposed Medline search strategy.

We will further search the reference lists of included studies and relevant systematic reviews for studies meeting our inclusion criteria. Further, to identify the most recent data, we will (1) search international clinical trial registers for recently completed trials, including the Australian and New Zealand Clinical Trials Registry, European Union Clinical Trials Register (EudraCT), International Standard Randomised Controlled Trial Number, US Clinical Trials Register, and the WHO Portal, and make attempts to identify published studies (e.g., contacting authors); (2) we will use Scopus and Web of Science to undertake forward citation searches for studies which fit our inclusion criteria; we will use the studies identified in our primary search and selection criteria, above, as the base of our forward citation search; (3) we will run our search strategy prior to submission of our manuscript for publication.

### Data management and extraction

Data will be managed during the screening and data extraction process using Covidence (https://www. covidence. org) and Microsoft Excel. Two review authors will independently screen the titles and abstracts of search results for relevance; we will acquire full texts of relevant articles, and two review authors will then screen these for relevance independently. Where full text cannot be acquired, we will contact the authors of relevant studies and request published or draft manuscripts. Throughout the process, a third author will assist in resolving screening conflicts.

Data, described in Table 1, will be extracted from relevant articles by two authors working independently. Data will be extracted into a study-specific database (Microsoft Excel file), and we will include a data dictionary and instructions for extracting researchers. Extracting researchers will receive training and pilot the database with a minimum of four studies each prior to formal extraction. Discrepancies will be resolved by adjudication by raters after any necessary recoding.

### Risk of bias

Two authors will independently assess study quality, via risk of bias, in the studies, per the PEDro scale [49]. The use of the PEDro tool will (1) cement comparison between pre-2014 and contemporary studies and (2) allow an overall narrative comment on reporting and study quality in studies reporting safety data. This data will be entered into the aforementioned database. Descriptive domains in the PEDro tool assess the external validity of the results (i.e., eligibility criteria) and the quality of the trial (i.e., randomisation, concealed group allocation, baseline similarities of groups, subject blinding, therapist blinding, assessor blinding, key outcomes attained from 85% of the sample, intention to treat analysis, statistics compared for at least one key outcome, and statistics compared at more than one time-point). Consistent with the literature on the use of the PEDro rating mechanism [49], we will score each study on 10 of the 11 criteria, with the criterion involving eligibility criteria not being included in the overall score. We will categorise higher quality studies as studies with a PEDro score of ≥ 6 and lower quality studies as studies with a PEDro score of <6 [49]. We will judge the rater agreement on study quality with Kappa [50]. Discrepancies will be resolved by adjudication by raters after any necessary recoding.

### Outcomes

The primary outcomes of interest for the review are (1) relapse, (2) AE, and (3) SAE. Secondary outcomes are (4) dropout/retention and (5) adherence/compliance. We define all terms in Table 1.

Per group, we will quantify the number of relapses and, separately, the number and type of AE and SAE. Reporting AE and adverse effects together will allow comparison between previous results [35] and those reported in studies published after November 2013. We will provide a descriptive analysis and narrative description of adverse effects and serious adverse effects where possible. Similarly, we will report retention as the number of completing participants per groups. We will provide descriptive data on adherence and compliance to the intervention, in the intervention groups only.

We will undertake a narrative synthesis and critical appraisal based on the risk of bias per PEDro scores, and we will make a narrative comment on the difference in safety reporting based on study quality. We will further consider a narrative synthesis of participant characteristics (e.g., type of MS and years since diagnosis) to describe and identify the clinical and policy implications of our findings.

### Analysis

Analyses will be conducted using SPSS (IBM SPSS v24; *MeanES and MetaReg* macros) and Stata (Stata Corp v14; *meta*).

#### Calculation of relative risk

Overall, we will calculate the relative risk of each of our three primary outcomes and retention into the study compared with control conditions. The unit of analysis for each of our three outcomes is the total number of relapses, adverse events, or serious adverse events experienced in each group of each study. For example, the overall rate of relapse, defined as the total number of reported relapses in the exercise and control conditions (i.e., number of relapses in each condition by the total number in each condition), will be pooled across all studies, respectively. We will calculate the overall relative risk of relapse using standard risk procedures, that is a ratio of rates, or the ratio of participants in a treatment group who experience an illness, condition, or event (i.e., relapse) to those in a control group who experience the same illness, condition, or event [51]. We will calculate relative risk by dividing the overall rate of relapse, AEs, SAEs, or dropout for exercise by the overall rate of relapse, AEs, SAEs, or dropout, respectively, in the control condition. A relative risk of 1.0 would indicate no difference in risk of the outcome between exercise and control conditions. A relative risk above 1.0 would indicate a higher risk of events with exercise training, and a relative risk below 1.0 would indicate a lower risk of events with exercise training. We will only report data from studies that directly report on the presence or absence of relapse or events. We will use standard procedures [52] to calculate relative risk, its standard error, and 95% confidence interval. Where zeros create problems with computation of relative risk or standard errors, we will add 0.5 to all cells (e.g., exercise relapse, exercise non-relapse, control relapse, and control non-relapse) [53, 54].

#### Data synthesis and analysis

Using an SPSS *MeanES* macro [55], a random-effects model will be used to aggregate mean relative risk for each of the primary outcomes. Based on established methods [56], we will calculate Cochrane *Q* and *I*^2^ to evaluate heterogeneity and consistency, respectively. In addition, sampling error will be calculated using Cochrane’s *Q* according to established methods; heterogeneity will be indicated if sampling error accounts for less than 75% of the observed variance and when *p*<0.05 for *Q* [57]. *I*^2^ values of 25%, 50%, and 75% will indicate small, moderate, and large amounts of heterogeneity, respectively [51]. Forest plots will be generated to illustrate the distribution of each effect size weighted by its inverse variance. Funnel plots will be generated and visually inspected, and Egger’s regression [58] and Begg’s rank correlation will be conducted to examine risk of publication bias [58]. The number needed to treat for an additional harmful outcome (NNTH), expressed as a function of absolute risk, will be calculated to express the number of participants engaged in exercise training for which one additional adverse outcome (i.e., relapse, AE, SAE, or dropout) may be expected [59].

Random-effects models will be used to calculate the mean relative risk and 95% CIs for each level of moderator variables [55] or potential sources of variability in the mean relative risk that are of logical, theoretical, and/or prior empirical relation to exercise training among persons with MS. Where data permits (i.e., where there are three or more effects for levels of the moderator variable), moderators will be included in random-effects univariate meta-regression analysis with maximum likelihood estimation using the SPSS macro *MetaReg* [55]. Meta-regression offers the benefit of concurrent estimates of independent effects by multiple moderators on the variation in relative risk across trials. Tests of the regression model (*Q*_*R*_) and its residual error (*Q*_*E*_) will be reported. If meta-analysis is not possible for a given outcome based on fewer than three effects, a narrative synthesis of included study results will be performed that appraises reported outcomes, study characteristics and methodological procedures which may have contributed to reported results, and the quality of included studies.

## Discussion

Our updated review of the safety of exercise interventions in MS will pool relapse, AE, and SAE events in RCTs published since November 2013. Since 2013, there has been a dramatic increase in the number of clinical exercise studies in MS [9] and a change in study design, for example, exercise intervention type (e.g., vigorous intensity interval training) [37] and intervention delivery (e.g., remotely supervised exercise training). This highlights a need to further clarify safety. In addition, since the publication of minimal exercise guidelines for exercise in MS in 2013 [19, 29], there is a need to make comment on the comparative safety reporting in studies prescribing these guidelines. Focal review of interventions delivering the exercise guidelines has not been undertaken; this is fundamental to the clinical promotion of these guidelines. We will compare previous literature on exercise training safety in MS [35–37], and with our planned review of retention rates, intervention adherence rates, and intervention compliance rates, we will confirm recent meta-analyses [40]. Our protocol provides a systematic and robust approach to determining the safety of exercise training in a clinical population and has high utility for repetition in other clinical populations such as stroke, Parkinson’s disease, and other populations where exercise prescription is a management strategy.

This review will be the first to consider exercise training safety when comparing participant characteristics (i.e., disability level). Identification of exercise training safety will be informative for persons with MS, for clinicians, and MS advocacy groups when providing lifestyle behaviour recommendations, for clinicians and researchers when considering intervention delivery and design, and for funding bodies and policy-makers when considering the impact of research applications.

Limitation of this systematic review and meta-analysis include the potential heterogeneity of research methodology that we expect to find in the included studies which will impact our choice of data analysis. In addition, reporting of outcomes may limit possible analyses. Where our planned analyses are inappropriate, we will proceed with narrative synthesis. We will report any deviation to the protocol in the full review.

## Supplementary Information

**Additional file 1:.** Supplementary material

## Data Availability

Not applicable

## Abbreviations

AE: Adverse event
MS: Multiple sclerosis
PEDro: Physiotherapy Evidence Database risk of bias
RCT: Randomised controlled trial
SAE: Serious adverse event
