# Supplementary material for: Safety of exercise training in multiple sclerosis: a protocol for an updated systematic review and meta-analysis
Source: Syst Rev. 2021 Jul 20;10:208. doi: 10.1186/s13643-021-01751-0 (PMC8293520; doi:10.1186/s13643-021-01751-0)
Supplement: Supplementary file 1 — Additional file 1:. Supplementary material [file 13643_2021_1751_MOESM1_ESM.docx]

**Supplementary material**

**Search strategies**

**Ovid Medline All**

(exercis* or movement).af.

1. ((exercis* or movement or (fitness or aerobic* or resistance or strength or endur*)) adj5 train*).af.
2. (physical* adj5 (activit* or therap* or rehabilitat* or endur* or stimulat* or educat* or medic*)).af.
3. occupational therap*.af.
4. (multiple sclerosis or disseminated sclerosis).af.
5. exp Exercise/ or exp Physical Therapy Modalities/ or exp Physical Endurance/ or exp Physical Fitness/ or exp Rehabilitation/
6. exp Multiple Sclerosis/
7. 1 or 2 or 3 or 4
8. (therap* or recovery of function).af.
9. 8 and 9
10. 6 or 10
11. 5 or 7
12. 11 and 12
13. clinical trial.mp. or clinical trial.pt. or random:.mp. or tu.xs.
14. 13 and 14
15. limit 15 to yr="2020 -Current"

**Ovid EMBASE**

1. (exercis* or movement).af.
2. ((exercis* or movement or (fitness or aerobic* or resistance or strength or endur*)) adj5 train*).af.
3. (physical* adj5 (activit* or therap* or rehabilitat* or endur* or stimulat* or educat* or medic*)).af.
4. occupational therap*.af.
5. (multiple sclerosis or disseminated sclerosis).af.
6. exp exercise/ OR physiotherapy / OR endurance/ OR endurance training/ OR training/ OR exp physical activity/ OR rehabilitation/ OR fitness/
7. multiple sclerosis/
8. 1 or 2 or 3 or 4
9. (therap* or recovery of function).af.
10. 8 and 9
11. 6 or 10
12. 5 or 7
13. 11 and 12
14. random:.tw. OR clinical trial:.mp. OR exp health care quality
15. 13 and 14
16. limit 15 to yr="2020 -Current"

**ProQuest PsycINFO**

1. exercis* OR movement
2. (exercis* OR movement OR fitness OR aerobic* OR resistance OR strength OR endur*) NEAR/5 train*
3. physical* NEAR/5 (activit* OR therap* OR rehabilitat* OR endur* OR stimulat* OR educat* OR medic*)
4. “occupational therap*”
5. “multiple sclerosis” OR “disseminated sclerosis”
6. therap* OR “recovery of function”
7. 1 OR 2 OR 3 OR 4
8. 6 AND 7
9. MAINSUBJECT.EXACT("Physical Fitness") OR MAINSUBJECT.EXACT("Physical Endurance") OR MAINSUBJECT.EXACT("Physical Therapy") OR MAINSUBJECT.EXACT("Physical Treatment Methods") OR MAINSUBJECT.EXACT.EXPLODE("Exercise") OR MAINSUBJECT.EXACT("Rehabilitation")
10. MAINSUBJECT.EXACT("Multiple Sclerosis")
11. 8 OR 9
12. 5 OR 10
13. 11 AND 12
14. (("random* control*" OR clinical) PRE/1 trial*) OR MAINSUBJECT.EXACT.EXPLODE("Clinical Trials")
15. 13 AND 14
16. Applied filters: 2013-11-01 - 2020-03-05

**Ebscohost CINAHL**

exercis* OR movement

1. (exercis* OR movement OR fitness OR aerobic* OR resistance OR strength OR endur*) N5 train*
2. physical* N5 (activit* OR therap* OR rehabilitat* OR endur* OR stimulat* OR educat* OR medic*)
3. “occupational therap*”
4. “multiple sclerosis” OR “disseminated sclerosis”
5. therap* OR “recovery of function”
6. 1 OR 2 OR 3 OR 4
7. 6 AND 7
8. (MH "Physical Fitness+") OR (MH "Physical Therapy+") OR (MH "Rehabilitation") OR (MH "Therapeutic Exercise+") OR (MH "Exercise+") OR (MH "Physical Endurance+")
9. (MH "Multiple Sclerosis")
10. 8 OR 9
11. 5 OR 10
12. 11 AND 12
13. (("random* control*" OR clinical) W1 trial*) OR (MH "Clinical Trials+")
14. 13 AND 14
15. Limiters - Published Date: 20130101-20201231

**Cochrane Library**

1. exercis* OR movement
2. (exercis* OR movement OR fitness OR aerobic* OR resistance OR strength OR endur*) NEAR/5 train*
3. physical* NEAR/5 (activit* OR therap* OR rehabilitat* OR endur* OR stimulat* OR educat* OR medic*)
4. occupational NEXT therap*
5. “multiple sclerosis” OR “disseminated sclerosis”
6. therap* OR “recovery of function”
7. 1 OR 2 OR 3 OR 4
8. 5 AND 6 AND 7
9. with Publication Year from 2013 to 2020, with Cochrane Library publication date from Nov 2013 to Feb 2020, in Trials

**Pedro**

Search 1

Abstract & Title: “multiple sclerosis” exercis* therap*

Method: clinical trials

Published Since: 2013

Search 2 (=101)

Abstract & Title: “multiple sclerosis”

Therapy: fitness training

Method: clinical trials

Published Since: 2013

Search 3 (=70)

Abstract & Title: “multiple sclerosis”

Therapy: strength training

Method: clinical trials

Published Since: 2013

**Scopus**

ALL( ( (exercis* OR movement ) OR ( ( (exercis* OR movement ) OR ( fitness OR aerobic* OR resistance OR strength OR endur* ) ) W/5 train* ) OR ( physical* W/5 ( activit* OR therap* OR rehabilitat* OR endur* OR stimulat* OR educat* OR medic* ) ) OR ( "occupational therap*" ) ) AND ( "multiple sclerosis" OR "disseminated sclerosis" ) AND ( therap* OR "recovery of function" ) AND ( ( "random* control*" OR clinical ) PRE/1 trial* ) ) AND NOT INDEX ( medline ) AND ( LIMIT-TO ( PUBYEAR , 2020 ) OR LIMIT-TO ( PUBYEAR , 2019 ) OR LIMIT-TO ( PUBYEAR , 2018 ) OR LIMIT-TO ( PUBYEAR , 2017 ) OR LIMIT-TO ( PUBYEAR , 2016 ) OR LIMIT-TO ( PUBYEAR , 2015 ) OR LIMIT-TO ( PUBYEAR , 2014 ) OR LIMIT-TO ( PUBYEAR , 2013 ) )

**Web of Science**

1. exercis* OR movement
2. (exercis* OR movement OR fitness OR aerobic* OR resistance OR strength OR endur*) AND train*
3. physical* AND (activit* OR therap* OR rehabilitat* OR endur* OR stimulat* OR educat* OR medic*)
4. “occupational therap*”
5. “multiple sclerosis” OR “disseminated sclerosis”
6. therap* OR “recovery of function”
7. 1 OR 2 OR 3 OR 4
8. 5 AND 6 AND 7
9. ("random* control*" OR clinical) AND trial*
10. 8 AND 9
11. Refined by: PUBLICATION YEARS: ( 2020 OR 2019 OR 2018 OR 2017 OR 2016 OR 2015 OR 2014 OR 2013 )
